# Harm perceptions of waterpipe tobacco smoking among university students in five Eastern Mediterranean Region countries: A cross-sectional study

**DOI:** 10.18332/tid/89966

**Published:** 2018-05-15

**Authors:** Niveen M.E. Abu-Rmeileh, Ola Alkhuffash, Khalid Kheirallah, Aya Mostafa, Muhammad Darawad, Yahya Al-Farsi, Afzalhussein Yusufali, Justin Thomas, Mohamed Salama, Randah R. Hamadeh, Rima Nakkash, Ramzi G. Salloum

**Affiliations:** 1 Institute of Community and Public Health, Birzeit University, Occupied Palestinian Territories; 2 Statistic Department, Hacettepe University, Ankara, Turkey; 3 Department of Public Health, Medical School, Jordan University of Science and Technology, Irbid, Jordan; 4 Department of Community Environmental and Occupational Medicine, Faculty of Medicine, Ain Shams University, Cairo, Egypt; 5 The University of Jordan, Amman, Jordan; 6 Sultan Qaboos University, Muscat, Oman; 7 Dubai Medical College, Dubai, United Arab Emirates; 8 Zayed University, Abu Dhabi, United Arab Emirates; 9 Mansoura University, Mansoura, Egypt; 10 College of Medicine and Medical Sciences, Arabian Gulf University, Manama, Bahrain; 11 Faculty of Health Sciences, American University of Beirut, Beirut, Lebanon; 12 Department of Health Outcomes and Policy, College of Medicine, University of Florida, Gainesville, Florida, United States

**Keywords:** waterpipe, Eastern Mediterranean, harm perception, university students

## Abstract

**INTRODUCTION:**

Waterpipe tobacco smoking (WTS) continues to be very common in the Eastern Mediterranean Region (EMR), partially because of cultural acceptance but also because of misconceptions of its harm. This paper aimed to describe the beliefs towards waterpipe harm of university students who smoked waterpipe in five EMR countries.

**METHODS:**

This study was conducted in 2016 across five EMR countries: Egypt, Jordan, Occupied Palestinian Territories, Oman and United Arab Emirates (UAE). Participants were recruited from among university students in each country. Students’ characteristics, smoking behavior, flavor preference and knowledge of WTS harm were collected using an internet-based survey. Participants were included if they were ever waterpipe tobacco smokers and between 18 and 29 years of age. Bivariate analyses assessed variations in student-perceived WTS harm across the countries. Linear regression analysis was used to assess WTS perceived harm differences between students in the different countries.

**RESULTS:**

A total of 2 544 university students participated from the five countries. Among ever smoking students, 66% reported WTS in the past 30 days, with the highest proportions (40%) from Occupied Palestinian Territories (OPT) and (41%) Jordan. Dual smoking of waterpipe and cigarettes was highest among students from Egypt. Most participants from the five countries had high level of perceived harm related to WTS during pregnancy. Less than 50% of the students believed that WTS could lead to the death of the smoker, can be harmful for non-smokers and have an addictive effect. Female students, those older than 22 years, and those who didn’t smoke waterpipe in the last 30 days significantly had a higher level of WTS perceived harm. Participating students believed that cigarettes are more addictive and contain more nicotine compared to waterpipe.

**CONCLUSIONS:**

Misperceptions of waterpipe harm are common among university students in the five EMR countries. Immediate public health action is needed, including enforcement of waterpipe tobacco control regulations along with awareness campaigns.

## INTRODUCTION

The Eastern Mediterranean Region (EMR) has some of the highest prevalence rates of waterpipe tobacco smoking (WTS) and these rates have been increasing over the past two decades^1-8^. In this region, it is culturally more acceptable for young people to smoke waterpipe than cigarettes. In fact, sharing waterpipe with family members is often how WTS is initiated among the young^9-11^. In most EMR countries, prevalence rates of WTS are slightly higher in boys. The gender gap in prevalence rates is much larger, however, for cigarette smoking^12,13^. Such gender differences were attributed to the social acceptability of WTS, especially among girls, compared to cigarette smoking^14^. Some studies have shown that, among youths in most of the EMR, waterpipe has replaced cigarettes as the most common method of tobacco use^15^.

A review of attitude, beliefs and perceived WTS harm has indicated that waterpipe smokers perceive it to be less harmful than cigarettes and so more likely to continue to smoke waterpipe^16^. These studies were limited to few constructs and they have not been compared across EMR countries that varied in WTS prevalence. Given the continuous increase in WTS, especially among young people in the Eastern Mediterranean countries and the limited studies, we aimed to assess the level of knowledge of WTS harm among university students in five EMR countries.

## METHODS

A cross-sectional study was conducted in 2016 across five EMR countries: Egypt, Jordan, Occupied Palestinian Territories, the United Arab Emirates (UAE) and Oman. The target population for the study was ever waterpipe smokers among young adults (18–29 years old) enrolled in universities in the selected countries. The participating universities were: Birzeit University (13 963), Jordan University of Science and Technology (20 000), University of Jordan (37 692), Sultan Qaboos University (15 357), Dubai Medical College (500), Zayed University (9 217), Mansoura University (91 041) and Ain Shams University (168 970).

Subjects were selected using convenience samples, with the purpose of recruiting relatively heterogeneous samples involving participants from key population groups (i.e. undergraduate vs graduate, and male vs female). Initial invitations were sent to all students through e-mail, Facebook, and students’ university portals inviting them to participate in the WTS survey with the aim of reaching a minimum of 400 students from each country. The estimated sample size for a proportion sample of a finite population with 95% Confidence Interval (CI) and assumption of 50% prevalence was 384 students. If the target size of 400 respondents was reached at a site before the end of the project, data collection continued to increase the sample size and hence power.

The email messages provided links to the internet-based survey. Participants were assured of the confidentiality of the survey, that their responses would remain anonymous and that their smoking status would not be made public, and finally were informed that they could leave the survey at any time.

The questionnaire used was adopted from a standard survey of university-based WTS users and measured basic demographic characteristics, WTS history, current use, attitudes and perceptions regarding WTS, and the concurrent use of other tobacco products. The health warning labels were pretested in qualitative interviews and the questionnaires were pre-piloted in each country. The questionnaire was translated into Arabic by two translators, and then reviewed and revised for nuances in dialect by the reseach team in each country.

Current waterpipe smokers and current cigarette smokers were defined as those who smoked in the last 30 days. Age, gender, WTS initiation location and perceived harm knowledge variables were measured for each country. Perceptions of harm were measured using 9 questions based on the following health warnings: WTS is addictive; waterpipe smoke can harm children; WTS causes fatal lung disease; WTS causes cancer; WTS causes strokes and heart disease; WTS during pregnancy can harm the baby; WTS can kill you; waterpipe smoke causes fatal lung disease in non-smokers; and quitting WTS now greatly reduces serious risks to your health. Each question had answers that were grouped into four codes: ‘Not at all/Little’ (1), ‘Somewhat’ (2), ‘A lot/Completely’ (3), and ‘Don’t know’ (4). For each participant, mean perceived harm was calculated using the nine above questions. The distribution of the WTS harm was tested for normality. Data were presented using numbers/percentages and means/SD as a appropriate.

Percentages and 95% CI were calculated for each variable mentioned above. Linear regression was used to assess variations in WTS level of harm beliefs between countries after adjusting for age and gender. For all analyses, we defined the level of significance at 0.05. The statistical analysis was conducted using SPSS version 22. Institutional Review Board approval was obtained from all participating institutions.

## RESULTS

A total of 2 544 university students participated: Jordan (745, 29.3%), OPT (772, 30.3%), Egypt (728, 28.6%), UAE (180, 7.1%) and Oman (119, 4.7%). Thirty-four per cent of all participants were females. Participants’ age ranged between 18 and 29 years, with a mean (SD) of 21.7 (2.8) years. Significant differences in the age and gender by country were detected (for both comparisons p<0.001) (Table 1).

**Table 1:** Study sample characteristics, in percentages (%)

	*Egypt (N=728 )*	*Jordan (N=745 )*	*OPT (N=772 )*	*Oman (N=119 )*	*UAE (N=180 )*	*Total (N=2544 )*
**Gender**						
Male	88.6	63.3	48.6	91.0	50.8	65.6
	(86.0–91.1)	(59.8–66.7)	(45.1–52.2)	(85.7–96.3)	(42.2–59.3)	(63.6–67.5)
Female	11.4	36.7	51.4	9.0	49.2	34.4
	(8.9–14.0)	(33.3–40.2)	(47.8–54.9)	(3.7–14.3)	(40.7–57.8)	(32.5–36.4)
**Age**
Young (18-22 years)	30.8	76.9	84.1	34.5	59.4	62.7
	(27.4–34.1)	(73.9–79.9)	(81.5–86.6)	(25.9–43.0)	(52.3–66.6)	(60.8–64.5)
Old (23 years or more)	69.2	23.1	15.9	65.5	40.6	37.3
	(65.9–72.6)	(20.1–26.1)	(13.4–18.5)	(57.0–74.1)	(33.4–47.7)	(35.5–39.2)
Mean	23.8 (2.86)	20.9 (2.20)	20.6 (2.19)	23.6 (2.93)	20.8 (2.45)	21.7 (2.80)
**Marital status**
Single	77.9	96.9	94.9	75.0	94.4	90.2
	(74.6–81.2)	(95.6–98.1)	(93.3–96.4)	(67.1–82.9)	(91.0–97.8)	(89.0–91.4)
Married	22.1	3.1	5.1	25.0	5.6	9.8
	(18.8–25.4)	(1.9–4.4)	(3.6–6.7)	(17.1–32.9)	(2.2–9.0)	(8.6–11.0)
**Employment status**
Unemployed	42.1	73.6	69.1	54.5	73.5	63.3
	(37.9–46.3)	(70.3–76.9)	(65.6–72.6)	(45.2–63.9)	(66.0–81.0)	(61.2–65.3)
Employed	57.9	26.4	30.9	45.5	26.5	36.7
	(53.7–62.1)	(23.1–29.7)	(27.4–34.4)	(36.1–54.8)	(19.0–34.0)	(34.7–38.8)
**Current waterpipe smoker (smoke in the past 30 days)**

No	26.2	31.5	36.8	46.1	47.8	33.8
	(22.7–29.7)	(28.1–34.8)	(33.3–40.2)	(37.0–55.2)	(40.4–55.1)	(31.9–35.7)
Yes	73.8	68.5	63.2	53.9	52.2	66.2
	(70.3–77.3)	(65.2–71.9)	(59.8–66.7)	(44.8–63.0)	(44.9–59.6)	(64.3–68.1)
**With whom did you first smoke waterpipe?**
Alone/no one	21.6	6.9	5.0	7.1	3.5	10.1
	(18.4–24.8)	(5.0–8.8)	(3.4–6.6)	(2.4–11.8)	(0.8–6.3)	(8.9–11.3)
With a friend	42.7	34.3	30.3	54.0	32.2	36.2
	(38.9–46.6)	(30.8–37.9)	(26.9–33.6)	(44.8–63.2)	(25.2–39.2)	(34.2–38.1)
With several friends	34.9	40.5	33.8	34.5	49.7	37.3
	(31.2–38.6)	(36.9–44.2)	(30.3–37.2)	(25.7–43.3)	(42.2–57.2)	(35.3–39.2)
With family members	0.8	18.2	31.0	4.4	14.6	16.5
	(0.1–1.5)	(15.4–21.1)	(27.6–34.3)	(0.6–8.2)	(9.3–19.9)	(15.0–18.0)
**Where did you first smoke waterpipe?**
Cafe/restaurant	81.4	51.8	39.9	74.3	72.3	58.9
	(78.4–84.4)	(48.1–55.6)	(36.3–43.5)	(65.7–82.8)	(65.5–79.1)	(56.9–60.9)
Smoke shop e.g. commercial waterpipe establishment	3.0	1.5	0.4	11.9	7.8	2.5
	(1.7–4.3)	(0.6–2.4)		(5.6–18.2)	(3.7–11.9)	(1.9–3.1)
At home	3.3	20.3	34.3	3.0	10.2	18.4
	(1.9–4.7)	(17.3–23.4)	(30.8–37.9)		(5.6–14.9)	(16.8–20.0)
Someone else's home	8.3	25.5	22.7	7.9	9.0	17.9
	(6.2–10.5)	(22.2–28.8)	(19.6–25.9)	(2.7–13.2)	(4.7–13.4)	(16.3–19.5)
University accommodations	3.9	0.9	2.6	3.0	0.6	2.3
	(2.4–5.4)	(0.2–1.6)	(1.4–3.7)			(1.7–2.9)

The majority of the students (74%) initiated WTS with friend(s), while 16.5% initiated it with a family member (Table 1). More than 70% of the students from Oman, UAE and Egypt started smoking in a café or restaurant, while around 50% of the students from Jordan and OPT started smoking at home or someone else’s home. Very few started WTS at university accommodations (Table 1).

**Figure 1:**
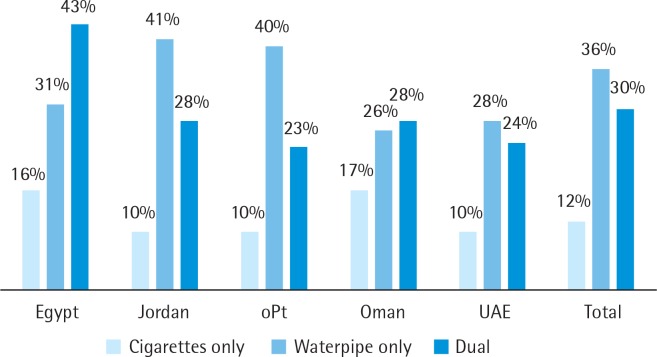
The percentages of current waterpipe tobacco smokers, current cigarette smokers and dual smokers, among university students in the EMR

Among waterpipe ever smokers, about two-thirds of participants reported WTS within the last 30-days (66.2%, 95% CI: 64.3–68.1%) (Figure 1). The overall percentage of current cigarette-only smokers was 12%, while the overall dual (waterpipe and cigarette) smoking percentage was 30%.

Table 2 presents university students’ perceived WTS harm among ever waterpipe smokers. Overall, 72% of all students believed that waterpipe smoking during pregnancy can harm the baby, 73% believed that WTS can be harmful to children, 62% believed that WTS can cause fatal lung disease and 60% believed that quitting WTS can reduce serious risk to health. Around 50% of all students believed that WTS causes cancer, stroke and heart diseases. Less than 50% of the students believed WTS causes fatal lung disease for non-smokers (46%), can kill (47%) and is addictive (30%). The answers of students from Egypt indicated the lowest level of perceived WTS harm and their answers were significantly different from those of students from the other four countries (Table 2).

**Table 2:** University students’ perceived WTS harm among ever waterpipe smokers in five EMR countries, in percentages (%)

	Egypt*	*Jordan*	*OPT*	*Oman*	*UAE*	*Total*
**Do you believe the following statements?**
**Waterpipe smoking is addictive**	**(N=620)**	**(N=739)**	**(N=758)**	**(N=116)**	**(N=178)**	**(N=2401)**
Not at all/Little	53.7	44.7	39.0	34.0	39.9	44.4
	(49.8–57.6)	(41.1–48.3)	(35.4–42.4)	(25.8–43.1)	(32.7–47.1)	(42.4–46.3)
Somewhat	21.8	25.4	23.4	24.1	23.6	23.6
	(18.5–25.0)	(22.2–28.5)	(20.3–26.4)	(16.4–31.9)	(17.4–29.8)	(21.9–25.3)
A Lot/Completely	23.2	27.4	36.4	41.4	35.4	30.4
	(19.9–26.5)	(24.2–30.7)	(33.0–39.8)	(32.4–50.3)	(28.4–42.4)	(28.6–32.3)
Don’t know	1.3	2.5	1.3	0	1.1	1.6
	(0.4–2.2)	(1.3–3.6)	(0.5–2.1)		(-0.4–2.7)	(1.1–2.1)
**Waterpipe smoke can harm children**	**(N=646)**	**(N=732)**	**(N=755)**	**(N=114)**	**(N=177)**	**(N=2424)**
Not at all/Little	24.5	10.7	6.2	15.8	10.7	13.2
	(21.1–27.8)	(8.4–12.9)	(4.5–7.9)	(9.1–22.5)	(6.2–15.3)	(11.9–14.5)
Somewhat	15.9	8.6	11.9	16.7	8.5	12.0
	(13.1–18.8)	(6.6–10.6)	(9.6–14.2)	(9.8–23.5)	(4.4–12.6)	(10.7–13.3)
A Lot/Completely	58.2	78.4	81.2	67.5	78.5	73.4
	(54.4–62.0)	(75.6–81.5)	(78.4–84.0)	(58.9–76.1)	(72.5–84.6)	(71.7–75.2)
Don’t know	1.4	2.2	0.73	0	2.3	1.4
	(0.5–2.3)	(1.1–3.2)	(0.1–1.2)		(014–4.4)	(0.9–1.9)
**Waterpipe smoking causes fatal lung disease**	**(N=633)**	**(N=735)**	**(N=756)**	**(N=114)**	**(N=178)**	**(N=2416)**
Not at all/Little	27.3	13.7	8.9	13.2	10.1	15.5
	(23.9–30.8)	(11.3–16.2)	(6.8–10.9)	(7.0–19.4)	(5.7–14.5)	(14.0–16.9)
Somewhat	20.4	16.5	19.7	25.4	15.2	18.8
	(17.2–23.5)	(13.8–19.1)	(16.9–22.5)	(17.4–33.4)	(9.9–20.4)	(17.3–20.4)
A Lot/Completely	49.0	66.4	68.0	61.4	68.0	62.3
	(45.1–52.9)	(63.0–69.8)	(65.1–71.7)	(52.5–70.3)	(61.1–74.8)	(60.4–64.3)
Don’t know	3.3	3.4	3.0	0	6.7	3.4
	(1.9–4.7)	(2.1–4.7)	(1.8–4.3)		(3.1–10.4)	(2.6–4.1)
**Waterpipe smoking causes cancer**	**(N=602)**	**(N=729)**	**(N=756)**	**(N=115)**	**(N=178)**	**(N=2380)**
Not at all/Little	25.1	16.2	16.0	25.2	11.2	18.4
	(21.6–28.5)	(13.5–18.9)	(13.4–18.6)	(17.3–33.2)	(6.6–15.9)	(16.9–20.0)
Somewhat	22.3	18.9	21.0	20.2	12.9	20.0
	(19.0–25.5)	(16.1–21.8)	(18.1–23.9)	(12.8–27.5)	(8.0–17.8)	(18.4–21.6)
A Lot/Completely	44.5	56.0	56.6	55.3	66.9	54.0
	(40.6–48.4)	(52.4–59.6)	(53.1–60.1)	(46.1–64.4)	(59.9–73.8)	(52.0–56.0)
Don’t know	8.1	8.9	6.3	0	9.0	7.5
	(6.0–10.3)	(6.8–11.0)	(4.6–8.1)		(4.8–13.2)	(6.4–8.5)
**Waterpipe smoking causes strokes and heart disease**	**(N=630)**	**(N=729)**	**(N=752)**	**(N=114)**	**(N=178)**	**(N=2403)**
Not at all/Little	33.2	15.0	16.0	24.6	15.2	20.5
	(29.5–36.9)	(12.4–17.5)	(13.3–18.6)	(16.7–32.5)	(1.6–28.7)	(18.9–22.1)
Somewhat	18.7	19.1	19.8	21.1	17.4	19.2
	(15.7–21.8)	(16.2–21.9)	(17.0–22.7)	(13.6–28.5)	(3.1–31.7)	(17.6–20.8)
A Lot/Completely	41.4	56.4	58.4	54.4	59.0	53.
	(37.6–45.3)	(52.8–60.0)	(54.9–61.9)	(45.2–63.5)	(40.4–77.5)	(51.2–55.2)
Don’t know	6.7	9.6	5.9	0	8.4	7.1
	(4.7–8.6)	(7.5–11.7)	(4.2–7.5)		(-2.1–18.9)	(6.1–8.1)
**Waterpipe smoking during pregnancy can harm the baby**	**(N=630)**	**(N=730)**	**(N=756)**	**(N=108)**	**(N=177)**	**(N=2401)**
Not at all/Little	22.9	10.0	5.8	16.7	7.3	12.2
	(19.6–26.1)	(7.8–12.2)	(4.2–7.5)	(9.6–23.7)	(3.5–11.2)	(10.9–13.5)
Somewhat	14.9	8.8	9.7	15.7	7.9	10.9
	(12.1–17.7)	(6.7–10.8)	(7.6–11.8)	(8.9–22.6)	(3.9–11.9)	(9.7–12.2)
A Lot/Completely	57.5	74.5	81.5	67.8	78.5	72.2
	(53.6–61.3)	(71.4–77.7)	(78.7–84.3)	(58.8–76.4)	(72.5–84.6)	(70.4–74.0)
Don’t know	4.8	6.7	3.0	0	6.2	4.7
	(3.1–6.4)	(4.9–8.5)	(1.8–4.3)		(2.7–9.8)	(3.9–5.6)
**Waterpipe smoking can kill you**	**(N=604)**	**(N=729)**	**(N=753)**	**(N=114)**	**(N=177)**	**(N=2377)**
Not at all/Little	37.9	22.6	24.3	21.9	21.5	26.9
	(34.0–41.8)	(19.6–25.7)	(21.2–27.4)	(14.3–29.5)	(15.4–27.5)	(25.1–28.7)
Somewhat	17.2	19.6	20.3	25.4	18.6	19.4
	(14.2–v20.2)	(16.7–22.5)	(17.4–23.2)	(17.4–33.4)	(12.9–24.4)	(17.8–21.0)
A Lot/Completely	39.6	48.6	48.3	52.6	52.6	46.7
	(35.7–43.5)	(44.9–52.2)	(44.8–51.9) )	(43.5–61.8)	(45.2–59.9)	(44.7–48.7)
Don’t know	5.3	9.2	7.0	0	7.3	6.9
	(3.5–7.1)	(7.1–11.3)	(5.2–8.9)		(3.5–7.1)	(5.9–8.0)
**Waterpipe smoke causes fatal lung disease in nonsmokers**	**(N=643)**	**(N=731)**	**(N=752)**	**(N=115)**	**(N=177)**	**(N=2418)**
Not at all/Little	35.8	21.1	24.1	20.9	22.0	26.0
	(32.1–39.5)	(18.1–24.0)	(21.0–27.1)	(13.4–28.3)	(15.9–28.1)	(24.2–27.7)
Somewhat	15.9	21.6	22.9	39.6	17.5	20.6
	(13.0–18.7)	(18.6–24.6)	(19.9–25.9)	(21.2–37.9)	(11.9–23.1)	(18.9–22.2)
A Lot/Completely	43.5	46.8	45.2	49.6	49.6	45.8
	(39.7–47.4	(43.2–50.4)	(41.7–48.8)	(40.4–58.7)	(42.9–57.6)	(43.8–47.8)
Don’t know	4.8	10.2	7.8	0	10.2	7.7
	(3.2–6.5)	(8.3–12.8)	(5.9–9.8)		(5.7–14.6)	(6.6–8.7)
**Quitting Waterpipe smoking now greatly reduces serious risks to your health**	**(N=643)**	**(N=730)**	**(N=754)**	**(N=113)**	**(N=179)**	**(N=2419)**
Not at all/Little	33.6	13.7	13.8	23.0	27.4	20.5
	(29.9–37.2)	(11.2–16.2)	(11.3–16.3)	(15.2–30.8)	(20.8–33.9)	(18.9–22.1)
Somewhat	14.0	16.8	16.9	20.9	14.7	15.6
	(11.3–16.7)	(12.3–17.5)	(14.2–19.5)	(13.4–28.4)	(9.5–19.9)	(14.1–17.0)
A Lot/Completely	48.8	66.1	66.8	54.5	52.5	60.3
	(45.0–52.7)	(62.6–69.5)	(63.5–70.2)	(45.6–64.0)	(45.2–59.9)	(58.3–62.2)
Don’t know	3.6	5.2	2.5	0	6.2	3.8
	(2.1–5.0)	(3.6–6.8)	(1.4–3.6)		(2.7–9.8)	(3.0–4.5)
**Mean perceived harm**	**(N=512)**	**(N=704)**	**(N=735)**	**(N=105)**	**(N=173)**	**(N=2229)**
	3.08	4.46	4.64	4.82	4.28	4.14
	(2.86-3.29)	(4.26-4.65)	(4.46-4.65)	(4.43-5.21)	(3.74-4.83)	(4.03-4.25)

* The total number was reported for each variable, as the number of missing was not consistent.

Table 3 presents the WTS harm for the five countries by gender, age and smoking status. Overall, female students indicated higher level of perceived WTS harm compared to male students. Only for the question on the effect of quitting smoking there was no significant gender difference. Further, answers from students older than 22 years indicated higher level of perceived WTS harm for the following questions: WTS can harm children, WTS causes fatal lung disease, WTS during pregnancy can harm the baby, and quitting WTS reduces risk to health. Finally, answers from current WTS users indicated lower level of perceived WTS harm compared to non-current smokers for all questions.

**Table 3:** University students’ perceived WTS harm among ever waterpipe smokers by selected factors, 2016, in percentages (%)

	*Gender*		*Current WTS*		*Age*	
	*Male*	*Female*	*No*	*Yes*	*18–22*	23+
**WTS is addictive**	**N(1486)**	**N(784)**	**N(776)**	**N(1557)**	**N(839)**	**N(1562)**
Not at all/Little	48.9	35.2	33.9	48.7	46.1	43.4
Somewhat	22.7	26.7	20.7	25.5	22.3	24.3
A lot/Completely	26.8	36.5	41.2	25.4	30.4	30.5
I don’t know	1.6	1.7	4.1	0.3	1.2	1.8
**Waterpipe smoke can harm children**	**N(1493)**	**N(786)**	**N(785)**	**N(1560)**	**N(863)**	**N(1561)**
Not at all/Little	12.2	7.3	10.3	12.1	22.2	8.2
Somewhat	13.6	9.2	8.3	13.8	13.9	10.9
A lot/Completely	72.4	82.8	79.9	72.8	62.7	79.4
I don’t know	1.8	0.8	1.5	1.3	1.2	1.5
**Waterpipe smoking causes fatal lung disease**	**N(1465)**	**N(785)**	**N(784)**	**N(1555)**	**N(853)**	**N(1563)**
Not at all/Little	14.4	10.5	11.0	15.2	23.6	11.1
Somewhat	21.0	16.3	11.6	23.1	19.9	18.4
A lot/Completely	61.2	69.8	74.6	57.9	53.6	67.1
I don’t know	3.4	3.4	2.8	3.7	2.9	3.6
**Waterpipe smoking causes cancer**	**N(1465)**	**N(785)**	**N(773)**	**N(1542)**	**N(821)**	**N(1559)**
Not at all/Little	18.2	14.4	12.9	19.5	23.3	15.9
Somewhat	20.7	20.9	14.2	23.4	19.9	20.1
A lot/Completely	53.0	57.7	66.1	49.1	51.4	55.4
I don’t know	8.1	7.0	6.7	8.0	5.5	8.5
**Waterpipe smoking causes strokes and heart disease**	**N(1477)**	**N(783)**	**N(778)**	**N(1545)**	**N(849)**	**N(1554)**
Not at all/Little	20.8	14.0	14.0	21.7	28.4	16.2
Somewhat	19.1	19.8	13.0	22.5	18.7	19.4
A lot/Completely	52.8	58.9	65.9	48.4	47.5	56.3
I don’t know	7.3	7.3	7.1	7.3	5.4	8.0
**Waterpipe smoking during pregnancy can harm the baby**	**N(1472)**	**N(786)**	**N(776)**	**N(1550)**	**N(843)**	**N(1558)**
Not at all/Little	11.0	6.2	10.3	10.5	20.5	7.6
Somewhat	11.8	9.0	7.6	12.5	13.5	9.6
A lot/Completely	71.1	82.2	78.6	71.7	62.0	77.7
I don’t know	6.1	2.5	3.5	5.4	3.9	5.1
**Waterpipe smoking can kill you**	**N(1463)**	**N(782)**	**N(766)**	**N(1544)**	**N(820)**	**N(1557)**
Not at all/Little	26.4	23.8	18.4	29.5	32.3	24.1
Somewhat	19.8	20.3	18.8	20.0	17.7	20.4
A lot/Completely	47.1	48.1	57.6	42.6	44.1	48.0
I don’t know	6.8	7.8	5.2	7.9	5.9	7.5
**Waterpipe smoke causes fatal lung disease in nonsmokers**	**N(1487)**	**N(784)**	**N(785)**	**N(1554)**	**N(862)**	**N(1556)**
Not at all/Little	26.0	19.5	19.5	27.2	30.6	23.4
Somewhat	20.8	22.2	17.2	22.7	18.8	21.5
A lot/Completely	44.9	51.1	56.9	41.7	45.7	45.9
I don’t know	8.3	7.1	6.4	8.4	4.9	9.2
**Quitting Waterpipe smoking now greatly reduces serious risks to your health**	**N(1487)**	**N(787)**	**N(784)**	**N(1554)**	**N(855)**	**N(1564)**
Not at all/Little	19.4	12.5	16.1	20.3	30.1	15.2
Somewhat	15.3	17.3	10.6	18.3	15.7	15.5
A lot/Completely	61.4	66.7	69.8	57.5	51.3	65.1
I don’t know	4.0	3.6	3.6	3.9	2.9	4.2
**Mean**	4.75	5.41	5.68	4.56	3.99	5.14
**(SD)**	(3.14)	(3.00)	(3.17)	(3.02)	(3.46)	(2.99)

**Figure 2:**
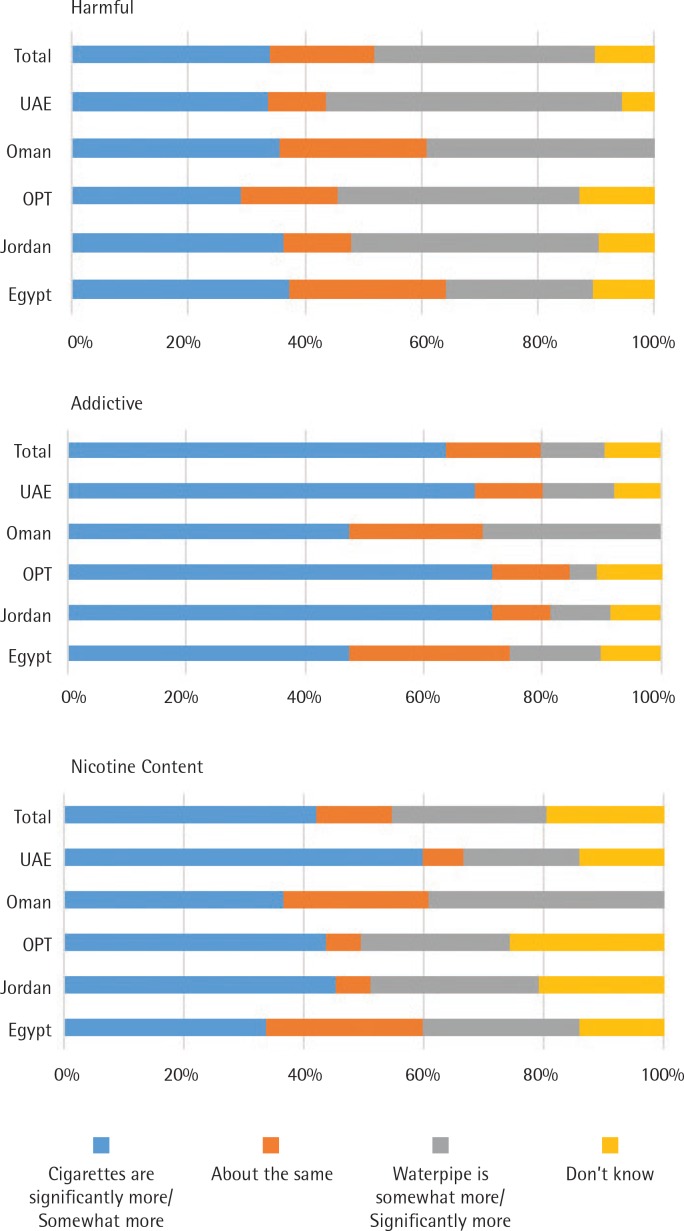
Perceived relative harm and addictiveness for waterpipe vs cigarettes among university students who are ever waterpipe smokers in five Eastern Mediterranean countries, 2016

When comparing WTS to cigarette, only 11% of all the students thought that WTS is more addictive compared to 64% who thought cigarette was more addictive. Seventy-two percent of the Jordanian and Palestinian students considered cigarettes more addictive than WTS. Less than a quarter of the students (26%) thought that WTS had more nicotine, while 42% thought that cigarettes had more nicotine. The UAE students compared to those from the other countries had the lowest percentage and thought that WTS had more nicotine compared to cigarettes. Students’ perceptions about the harmful effects of cigarettes and WTS were similar: 34% perceived cigarettes to be more harmful, and 37% perceived WTS to be more harmful. The lowest percentage (25%) was that for students from Egypt who thought WTS as less harmful than cigarette smoking, compared with other countries (Figure 2).

Mean harm perception scores were significantly higher among students from OPT (p<0.001), Jordan (p<0.001) and UAE (p=0.019) compared to students from Egypt and Oman after adjusting for age, gender and current WTS. Overall, current WTS students had significantly lower mean harm perception scores compared to non WTS students (p≤0.001).

## DISCUSSION

Despite increasing evidence on a WTS epidemic in the EMR, there is generally no parallel public health action taken to tackle this phenomenon^14^. The current study shows that university students in general had low knowledge of WTS harm, with misconceptions about the harmful and addictive effects of WTS. The level of knowledge on harm was lowest among the students from Egypt, despite several original research studies and systematic reviews documenting the harmful effects of WTS on health^17-20^.

An interesting finding was knowledge of the harmful effects that secondhand smoke from WTS has on children and on the fetus of women who smoke during pregnancy. Students perceived WTS as less harmful to themselves and to other non-smokers exposed to waterpipe. Another misconception was the belief that WTS is less addictive and contains less nicotine compared to cigarettes. In addition, the students’ belief was split between WTS was more harmful compared to cigarettes smoking, WTS had similar harm as cigarettes, and cigarettes were regarded as more harmful in all five countries. Increasing evidence supports that the harmful and addictive effects of WTS are similar to those of cigarette smoking^21-23^. The results of the current study confirm what was reported regarding perception and knowledge in a preliminary qualitative study that was conducted on EMR students through in-depth interviews^24^.

Egypt was found to have a uniquely high percentage of current smokers of waterpipe, cigarettes and dual, in addition to a high level of misconception of the harmful effects of waterpipe. These findings are consistent with prior studies conducted among different populations within Egypt that have indicated the high and increasing prevalence of WTS and the misconception of the harmful effects of waterpipe^25,26^.

There were variations across the five countries with respect to WTS initiation place and company. Jordanian and Palestinian students started WTS at home, whereas Omani, Emirati and Egyptian students more frequently initiated WTS at cafes or restaurants. The previous study explained the influence of smoking parents, especially on their children^27-29^, in addition to the influence of friends and peers^29,30^. Cultural acceptance might explain the high percentage of WTS compared to cigarettes smoking^31^, especially for women^32,33^. The Arab communities are more permissive toward WTS compared to cigarettes, even among conservative communities^27,34^.

Current tobacco policies in the selected countries are generally targeted toward cigarette control, which marginalizes control of other tobacco products including the waterpipe^35^. These countries have approved the World Health Organization Framework Convention on Tobacco Control (WHO FCTC), with the exception of OPT. Still, the enforcement of the WHO FCTC is low in most countries^36^ with more emphasis on text health warnings^37^. Thus, immediate action is needed, similar to cigarette control regulations, targeting bans on advertisement and sales to minors, taxation, warning labels, and smoke-free indoor air policies, to control WTS^38^.

Furthermore, health warning labels on tobacco packages serve as a prominent source of health information for smokers and non-smokers that increases health knowledge and perceptions of tobacco risks. The evidence indicates that comprehensive warnings, specifically pictorial health warnings, may help prevent smoking initiation^39^. The challenge with implementing health warnings for WTS is in its social nature, as it is served in cafes, resturants and homes, and consequently, the smoker does not have direct view of the tobacco packages and labels^40^. Hence, in addition to health warning labels on the waterpipe tobacco packages, labels need to be placed on waterpipe accessories^41^.

Currently, there is limited evidence on the most effective intervention for WTS prevention and cessation. Some studies are showing promising results, especially in increasing the level of knowledge about the harmful effects of WTS^42^. Behavioral cessation interventions based on evidence from cigarette smoking seems to be a good starting point. However, such interventions should take into consideration the cultural and social acceptance of WTS and its intermittent behaviour^38^.

### Strengths and limitations

This WTS study was conducted in five EMR countries using standardised methods on university students. The web-based survey might have some limitations, but it was the method of choice because it requires less time and effort. Further, the web-based surveys have been shown to yield similar response rates compared with more traditional survey modes, and perhaps higher response rates among university students. The study was based on convenience samples so findings may not be generalizable to a broader population. The sample was limited to ever users of WTS and future studies should focus on never users of WTS. The desired sample size was achieved for Egypt, Jordan and OPT, where waterpipe smoking prevalence is higher^43^ and smoking is more socially acceptable^24^ than in Oman and UAE.

## CONCLUSIONS

Misperceptions of waterpipe harm are common among university students in the five EMR countries. Immediate public health action is needed, including enforcement of waterpipe tobacco control regulations along with awareness campaigns.

## CONFLICTS OF INTEREST

Authors have completed and submitted the ICMJE Form for Disclosure of Potential Conflicts of Interest and none was reported.
